# The psychological distress and suicide-related ideation in hospital workers during the COVID-19 pandemic: Second results from repeated cross-sectional surveys

**DOI:** 10.1371/journal.pone.0277174

**Published:** 2022-11-10

**Authors:** Keiko Ide, Takeshi Asami, Akira Suda, Asuka Yoshimi, Junichi Fujita, Yohko Shiraishi, Munetaka Nomoto, Masatoshi Miyauchi, Tomohide Roppongi, Taku Furuno, Kaori Watanabe, Tomoko Shimada, Tomoko Kaneko, Yusuke Saigusa, Kazumi Kubota, Hideaki Kato, Toshinari Odawara, Akitoyo Hishimoto

**Affiliations:** 1 Department of Psychiatry, Yokohama City University Hospital, Yokohama, Japan; 2 Department of Psychiatry, Yokohama City University School of Medicine, Yokohama, Japan; 3 Health Management Office, Yokohama City University Hospital, Yokohama, Japan; 4 Department of Child Psychiatry, Yokohama City University Hospital, Yokohama, Japan; 5 Clinical Laboratory Department, Yokohama City University Hospital, Yokohama, Japan; 6 Psychiatric Center, Yokohama City University Medical Center, Yokohama, Japan; 7 Nursing Department, Yokohama City University Hospital, Yokohama, Japan; 8 Patient Care and Safety Management Department, Yokohama City University Hospital, Yokohama, Japan; 9 Nursing Department, Yokohama City University Medical Center, Yokohama, Japan; 10 Department of Biostatistics, Yokohama City University Graduate School of Medicine, Yokohama, Japan; 11 Department of Healthcare Information Management, The University of Tokyo Hospital, Tokyo, Japan; 12 Infection Prevention and Control Department, Yokohama City University Hospital, Yokohama, Japan; 13 Department of Hematology and Clinical Immunology, Yokohama City University School of Medicine, Yokohama, Japan; 14 Health Management Center, Yokohama City University, Yokohama, Japan; Al-Jouf University College of Pharmacy, SAUDI ARABIA

## Abstract

The COVID-19 pandemic has been affecting the mental health of hospital workers. During the prolonged pandemic, hospital workers may experience much more severe psychological distress, leading to an increased risk of suicide. This study aimed to investigate changes in psychological effects on hospital workers over 12 months from the beginning of the pandemic and clarify factors associated with psychological distress and suicide-related ideation 1-year after the pandemic’s beginning. These repeated, cross-sectional surveys collected demographic, mental health, and stress-related data from workers in 2 hospitals in Yokohama, Japan. The first survey, conducted in March-April 2020, contained the 12-item General Health Questionnaire (GHQ-12) assessing general distress and the Impact of Event Scale-Revised (IES-R) assessing event-related distress. In the second survey in March 2021, hospital workers at the same two hospitals were reassessed using the same questionnaire, and Item 9 of the Patient Health Questionnaire (PHQ-9) was added to assess their suicide-related ideation. The findings of the first and second surveys revealed that the average score of GHQ-12 (3.08 and 3.73, respectively), the IES-R total score (6.8 and 12.12, respectively), and the prevalence rates of severe general distress (35.0% and 44.0%, respectively) and severe event-related distress (7.0% and 17.1%, respectively) deteriorated. The second survey showed that 8.6% of the hospital workers were experiencing suicide-related ideation. Both the general and event-related distress were associated with suicide-related ideation. In these surveys, mental health outcomes among the hospital workers deteriorated over one year from the pandemic’s beginning, and their severe psychological distress was the risk factor for the suicide-related ideation. Further studies are needed to compare the psychological effects on hospital workers during and after the prolonged pandemic and to explore appropriate measures to support hospital workers’ mental health.

## Introduction

The spread of the novel coronavirus-2019 (COVID-19) pandemic has continued over the years, and the future is still uncertain. The COVID-19 pandemic has greatly affected public health and social structures, including interpersonal relationships. Moreover, it has been considered a significant threat due to invisible agents [1]. The multiple waves of the COVID-19 pandemic over the years required social and behavioral changes, leading to long-term anxiety and exhaustion and thus causing psychological distress in the long term. The psychological effects of the COVID-19 pandemic have been investigated not only in Asia, Europe and the United States, but also worldwide, targeting not only the general population but also various social groups, such as university students or people in specific regions [2–4].

Early in the COVID-19 pandemic, during a shutdown, health care workers (HCWs) put their safety on the line and kept working to care for patients [5]. In this prolonged pandemic, HCWs continue to battle the pandemic, inadequate support, escalating workloads, fear of infection, discrimination and so on. This complex combination of pressures increases the risk of adverse mental health outcomes for HCWs [6]. Mental health outcomes and associated factors among HCWs have already been reported in China, and many other countries worldwide [7–9].

Many experts agree that the COVID-19 pandemic affected the mental health of diverse populations with unpredictable increases in suicide rates [10, 11]. Although reasons for changes in suicide prevalence are extremely complex and association of suicide with the COVID-19 pandemic is unknown, the number of suicides in Japan had decreased for 10 consecutive years from 2010 until June 2020. The suicide rate in Japan declined by 14% during the first 5 months of the pandemic; however, it increased by 16% from July to October 2020 [12]. The increase was substantial, especially among women (37%) and younger age groups (49%).

Before pandemics, it has also been reported that HCWs are at increased risk for suicide compared to other occupational groups and the general public [13, 14]. In this pandemic, HCWs served on the front lines against COVID-19. They were described in heroic terms, although the ongoing pandemic-related stressors were expected to increase mental health concerns, including suicidality, among some HCWs [15]. It is essential to consider the effect of this disaster on public health; however, it is also critical to consider its long-term effects on HCWs [16, 17]. Recent studies have shown that the COVID-19 pandemic has heightened interest in physician mental health, and physicians are at an increased risk of suicide [18]. We hypothesized that the psychological distress of hospital workers during the prolonged pandemic might have deteriorated, and the hospital workers might have experienced much more severe psychological distress, leading to an increased risk of suicide.

Yokohama City University Hospital and Yokohama City University Medical Center are public university hospitals in Yokohama City, Japan. Since the large disease cluster occurred off the coast of Yokohama Port in February 2020 on the Diamond Princess cruise ship, these hospitals admitted COVID-19 patients in Japan’s early stage of the COVID-19 pandemic. The examination method, vaccination, or therapy drugs for COVID-19 had not been established at that time. The stigma and discrimination against COVID-19 responders might have been stronger in the early period; thus, hospital workers accepting COVID-19 patients might have experienced severe psychological distress.

We felt that hospital workers, both high-risk workers (e.g., working in the ward for COVID-19) and low-risk workers (e.g., working in the department without COVID-19 patients), experienced severe emotional distress. At that time, we conducted the first survey study with all our hospital workers at the University Hospital and the Medical Center and reported the results [19]. We aimed to clarify the psychological effect of the COVID-19 pandemic on hospital workers at the beginning of the pandemic and related factors.

One year after the pandemic, life had not returned to normal; moreover, Japan’s third wave of COVID-19 had come, and hospitalizations surged in January 2021. We then planned a second survey using the same methods because we hypothesized that the psychological distress of hospital workers during the prolonged pandemic might have deteriorated over 1-year from the beginning of the pandemic, and the hospital workers might have experienced much more severe psychological distress, leading to the increased risk of suicide. Although many cross-sectional and short-term longitudinal surveys have been designed to assess psychological effects during the early pandemic [7, 17, 20, 21], to our knowledge, long-term surveys on psychological effects, especially on hospital workers, have not been reported yet.

In this study, we aimed to investigate the changes in psychological effects and related factors throughout the COVID-19 pandemic by comparing the second survey with the first survey data. In addition, in this second survey, we aimed to clarify the psychological effect of the COVID-19 pandemic and suicide-related ideation on hospital workers 1-year after the pandemic’s beginning.

## Methods

### Study design and setting

A quantitative, descriptive, paper-based, repeated cross-sectional studies were conducted from March 23, 2020, to April 6, 2020, as the first survey and from March 8, 2021, to March 22, 2021, as the second survey. These surveys were conducted both in Yokohama City University Hospital and Yokohama City University Medical Center in Yokohama City, in Japan.

### Participants and data collection

Medical doctors, nurses, other medical professionals (including clinical laboratory technicians, radiological technologists, medical engineers, pharmacists, dieticians, social workers, physical therapists, occupational therapists, and speech therapists), office workers, clinical clerks, and other support staff (including nursing assistants, janitors, food service, and laundry staff) who worked in the University Hospital or the Medical Center in March 2020 in the first survey and in March 2021 in the second survey were invited to participate. The paper-based, self-administered anonymous questionnaires were handed to all hospital workers individually, placed on their desks, or delivered to their mailboxes. Subsequently, the in-house mail system collected them to ensure that all hospital workers responded to the surveys, including participants who did not have internet access or were unfamiliar with an online platform.

### Content of the questionnaire

The first question asked for informed consent to use the responses in the survey by checking the box. In the second survey, a question was added that asked whether they completed the first survey to count how many respondents filled out both surveys because the hospital workers during the first survey and the second survey might not have been the same participants due to the transfer, new employment or resignation, and the anonymous nature of the data collection, which would prevent us from pairing their data 1-year intervals.

The first survey consisted of four parts, sociodemographic questions, the 12-item General Health Questionnaire (GHQ-12) [22, 23], the Impact of Event Scale-Revised (IES-R) [24, 25], and stress-related questions associated with COVID-19. The second survey was identical to the first one, allowing us to compare the results longitudinally; however, Item 9 of the Patient Health Questionnaire (PHQ-9) [26–28] was added to the second survey to assess suicide-related ideation.

Sociodemographic question asked about age, gender, occupations, preexisting conditions, living with a partner, living with the elderly, confidence in standard precautions, and being in direct contact with at least one COVID-19 patient. These characteristics were assumed to relate to the risks of the COVID-19 infection.

We calculated total scores for the GHQ-12 using the GHQ scoring method [22] and subsequently divided our hospital workers into two groups, workers ‘with’ or ‘without’ severe general distress. A threshold of 3/4 was used because the mean scores of the GHQ-12 were 3.08 in the first survey and 3.73 in the second survey [29].

The IES-R is a self-report scale consisting of 22 items measured on a 5-point scale originally developed to assess posttraumatic stress syndrome, and it is used widely to assess the psychological response to a stressful event. The IES-R total comprises three subscales assessing major posttraumatic stress disorder symptoms: intrusion, avoidance, and hyperarousal. We used the IES-R to assess the ‘event-related distress of the COVID-19 pandemic.’ A score of 25 or more indicated a ‘high level of event-related distress’ [30–32].

Item 9 of the PHQ-9 asked, “Over the last two weeks, how often have you been bothered by thoughts that you would be better off dead or hurting yourself in some way?” The responses were rated on a 4-point Likert scale (0 = Not at all; 1 = Several days; 2 = More than half the days; 3 = Nearly every day). We used Item 9 of PHQ-9 to assess the ‘suicide-related ideation,’ and a score of 1 or more was treated as ‘suicide-related ideation,’ according to the previous literature [20, 33–35]. Previous analysis indicated that response to Item 9 of PHQ-9 identifies outpatients at increased risk for suicide attempt or death [28].

The 26 stress-related questions were scored using the GHQ scoring method [22] (never = 0; rarely = 0; sometimes = 1; always = 1), dividing our hospital workers ‘with’ or ‘without’ various stressors. Nineteen of the 26 items were adapted from studies on the SARS [36] and the H1N1 influenza pandemics [37], and seven original items were added to inquire about family support (Q11) and increased exposure to TV (Q22) and internet media (Q23). These 26 items were the same as in the previous survey. The Cronbach’s alpha coefficient for the 26 stress-related questions was α = 0.81, indicating good internal consistency and acceptable reliability.

Yokohama City University Ethical Review Board approved this study (B210100008, B210100013), and participation was voluntary.

### Statistical analysis

We conducted descriptive analyses to examine the participants’ characteristics and estimate the prevalence of general distress, event-related distress, and suicide-related ideation. Subsequently, we conducted chi-square tests to compare the two surveys on the prevalence of general distress, event-related distress, and suicide-related ideation, and to identify sociodemographic factors associated with severe general distress, event-related distress, and suicide-related ideation.

Next, we conducted a Mann-Whitney U test to compare the two surveys in the mean of GHQ-12 scores, means of the total IES-R, and each subscale score to assess whether hospital workers had much more severe psychological distress.

We conducted a factor analysis of the 26 stress-related questions to determine the synthetic variables using the maximum likelihood method and Promax rotation. The number of factors was determined by the size of the eigenvalue (greater than 1.00) and the relative size of the values according to different factor models. For each factor, the total scores of the stress-related questions were calculated.

In the first survey, we hypothesized that the low-risk workers might be psychologically affected differently compared to the high-risk workers because only a few patients with COVID-19 have been admitted to our hospitals since the outbreak began. Therefore, the first study conducted multivariable logistic regression analyses for high- and low-risk workers separately [19]. In the second survey, however, there were much more COVID-19 patients in our hospitals and the possibility of having contact with COVID-19 patients was much higher for not only the high-risk workers but also the low-risk workers. Hence, we speculated that we did not need to focus on the psychological difference between the high- and the low-risk workers.

Multivariable logistic regression analyses were conducted to determine potential risk factors for general and event-related distress and suicide-related ideation (forced entry method). ‘Workers with/without more severe general distress, as evaluated by the GHQ-12,’ ‘Workers with/without event-related distress, as assessed by the IES-R,’ or ‘Workers with/without suicide-related ideation, as assessed by Item 9 of the PHQ-9’ were used as dependent variables, and the demographic data and the total scores of the factors derived from the factor analysis (factors 1–7) were treated as independent variables. The association between risk factors and outcomes is presented as adjusted odds ratios (Adjusted ORs) and 95% CIs with the risk factors, which were gender, age group, occupation, preexisting disease, living with a partner, living with the elderly, confident in standard precaution, factors 1–7, and direct contact with COVID-19 patients. To assess associations between risk factors and event-related distress specific to the COVID-19 pandemic, severe general distress evaluated by the GHQ-12 was included as additional independent variables in the event-related distress models. Severe general distress and severe event-related distress assessed by the IES-R were used as additional independent variables in the suicide-related ideation model.

To compare the two surveys, multivariable logistic regression analyses were conducted using the results from the first survey. The demographic data and the total scores of the factors 1–7 derived from the factor analysis in the second survey and severe general distress added in the event-related distress model were treated as independent variables, like in the second survey. The result similar to Table 2 was obtained from the factor analysis in the same manner as the present paper applied to the data in the first survey (the details were not reported).

Data analyses were performed using the SPSS statistical software version 21.0 (IBM Corp) with a significance level set at p < .05 (two-tailed).

## Results

### Summary of the first study

The first survey was distributed to 4133 hospital workers, and 2697 (65.3%) valid questionnaires were used for analyses. Of all hospital workers, 944 (35.0%) showed general distress, and 189 (7.0%) demonstrated event-related distress. Multivariable logistic regression analyses revealed that the ‘Feeling of being isolated and discriminated’ factor was associated with the general and event-related distress for both the high- and low-risk workers. The ‘Exhaustion’ was also related to both the general and event-related distress of the low-risk workers and the general distress of the high-risk workers. The ‘Anxiety about infection,’ showed no association with the general or event-related distress of the high-risk workers; however, it demonstrated significant relationships with both types of distress among low-risk workers. In the first survey, both high-risk and low-risk workers reported experiencing psychological distress at the beginning of the outbreak.

### Descriptive analyses

In the second survey, a questionnaire was distributed to 4321 hospital workers, 2168 in the University Hospital and 2153 in the Medical Center, and 3109 completed the questionnaires (72.0%). Of these, 296 were excluded because the informed consent was not checked, or at least one answer was missing on sociodemographic characteristics or psychological rating scales. Therefore, 2813 questionnaires (65.1%) were used for our second analyses. Of these, 1548 respondents (55.0%) reported that they also completed the first survey; therefore, they were considered to complete both the first and second surveys.

The first column of Table 1 shows the characteristics of the second survey participants. Of the 2813 participants, 2078 (73.9%) were women, 1055 (37.5%) were nurses, and 588 (20.9%) were medical doctors. Furthermore, 1172 (41.7%) had a direct contact with COVID-19 patients (12.2% at the first survey), 1237 (44.0%) showed severe general distress (score of GHQ-12 = 4 or more), 480 (17.1%) demonstrated severe event-related distress (score of IES-R = 25 or more), and 243 (8.6%) had suicide-related ideation (score of Item 9 of PHQ-9 = 1 or more).

**Table 1:** Participants’ characteristics associated with severe general distress, severe event-related distress, and suicide-related ideation.

	overall	severe general distress ^a^		severe event-related distress ^b^		suicide-related ideation ^c^	
		n (%)	p-value	n (%)	p-value	n (%)	p-value
Total	2813	1237 (44.0%)		480(17.1%)		243(8.6%)	
Gender							
Men	735	259(35.2%)		102 (13.9%)		49(6.7%)	
Women	2078	978(47.1%)	< .001**	378(18.2%)	0.007*	194(9.3%)	.027*
Age, years							
29>	667	314(47.1%)		101(15.1%)		68(10.2%)	
30–39	727	302(41.5%)		98(13.5%)		76(10.5%)	
40–49	737	331(44.9%)		136(18.5%)		62(8.4%)	
50<	682	290(42.5%)	< .001**	145(21.3%)	< .001**	37(5.4%)	.003*
Occupation							
Medical Doctor	588	184(31.3%)		55(9.4%)		39(6.6%)	
Nurse	1055	527(50.0%)		182(17.3%)		91(8.6%)	
Other medical	390	185(47.4%)		68(17.4%)		41(10.5%)	
Office workers/ clinical clerks	548	251(45.8%)		119(21.7%)		51(9.3%)	
Other support staff	232	90(38.8%)	< .001**	56(24.1%)	< .001**	21(9.1%)	.278
Preexisting disease							
yes	264	121(45.8%)		57(21.6%)		22(8.3%)	
no	2549	1116(43.8%)	.523	423(16.6%)	.040*	221(8.7%)	.853
Living with partner							
yes	1539	620(40.3%)		242(15.7%)		105(6.8%)	
no	1274	617(48.4%)	< .001**	238(18.7%)	.039*	138(10.8%)	< .001**
Living with elderly							
yes	362	181(50.0%)		86(23.8%)		35(9.7%)	
no	2451	1056 (43.1%)	0.015*	394(16.1%)	.001*	208(8.5%)	.482
Confident in standard precaution							
yes	1790	748(41.8%)		283(15.8%)		134(7.5%)	
no / not answered	1023	489(47.8%)	0.002*	197(19.3%)	.022*	109(10.7%)	.005*
Direct contact with COVID-19 patient							
yes	1172	524(44.7%)		188(16.0%)		82(7.0%)	
no or don’t know	1641	713(43.4%)	.507	292(17.8%)	.223	161(9.8%)	.009*
Severe general distress							
GHQ-12≤3	1576	-		105(6.7%)		29(1.8%)	
4≤ GHQ-12	1237	-	-	375(30.3%)	< .001**	214(17.3%)	< .001**
Severe event-related distress							
IES-R≤24	2333	862(36.9%)		-		125(5.4%)	
25≤ IES-R	480	375(78.1%)	< .001**	-	-	118(24.6%)	< .001**

Abbreviations: GHQ-12, the 12-item General Health Questionnaire; IES-R, the impact of Event Scale-Revised

^a^ Severe general distress evaluated by the GHQ-12 (≥4).

^b^ Severe event-related distress evaluated by the IES-R (≥25).

^c^ suicide-related ideation evaluated by the score of Item 9 of PHQ-9 (≥ = 1).

Two-tailed chi-square tests were performed to evaluate differences in proportions (Table 1). Women were significantly more likely to report severe general distress (47.1%), severe event-related distress (18.2%), and suicide-related ideation (9.3%) than men (35.2%, 13.9%, and 6.7%, respectively). Among the age groups, employees in their 20s were most likely to have severe general distress (47.1%), while those in their 50s were most likely to have severe event-related distress (21.3%). Employees in their 30s and 20s were most likely to have suicide-related ideation (10.2% and 10.5%, respectively). Compared to the medical doctors, employees in other occupations reported significantly more severe general and event-related distress (e.g., severe general distress between medical doctors and nurses: 31.3% vs. 50.0%; severe event-related distress between medical doctors and other support staff: 9.4% vs. 24.1%).

Individuals with the preexisting disease were significantly more likely to report severe event-related distress (21.6% vs. 16.6%). We think that those with the preexisting disease might be anxious about infection and worsening of the COVID-19. Those living without partners were more likely to report severe general distress (48.4% vs. 40.3%) and suicide-related ideation (10.8% vs. 6.8%). We think that the availability of partners or families might have buffering effects on the distress despite their worries about passing infection. Participants living with the elderly were more likely to report severe general (50.0% vs. 43.1%) and event-related distress (23.8% vs. 16.1%). We think that those living with the elderly might fear about passing infection to their elder families and worsening the elderlies’ illness. Those with confidence in the standard precaution were less likely to report severe general distress (41.8% vs. 47.8%), severe event-related distress (15.8% vs. 19.3%), and suicide-related ideation (7.5% vs. 10.7%) than those without confidence. Furthermore, individuals without direct contact with COVID-19 patients did not show significant severe general or event-related distress; however, they had significantly higher suicide-related ideation rates (9.8%) compared to those with direct contact with at least one COVID-19 patient (7.0%). Severe general distress was associated with severe event-related distress (30.3% vs. 6.7%) and suicide-related ideation (17.3% vs. 1.8%). Moreover, severe event-related distress was associated with suicide-related ideation (24.6% vs. 5.4%).

### Time 1 and Time 2

**Fig 1:**
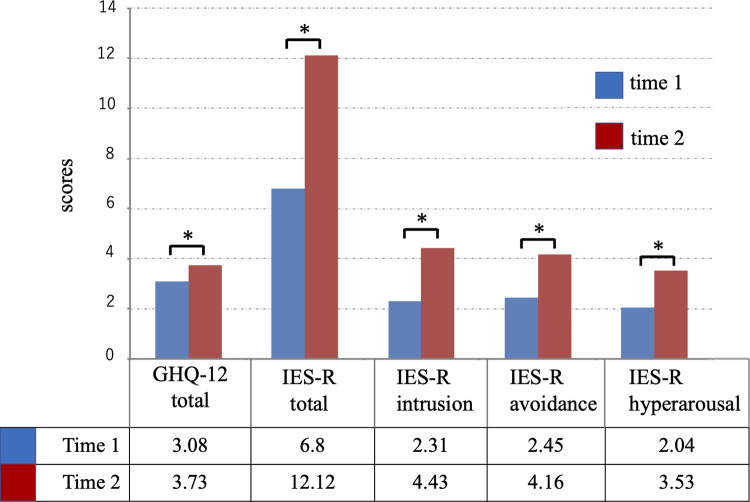
The GHQ-12 and the IES-R scores at Time 1 and Time 2. The average score of GHQ-12, IES-R total, and IES-R subscales, intrusion, avoidance, and hyperarousal at Time 2, were significantly higher than at Time 1. Abbreviations: GHQ-12, the 12-item General Health Questionnaire; IES-R, the impact of Event Scale-Revised; Time 1 = first survey (March-April 2020); Time 2 = second survey (March 2021). (*, p < .05, Mann-Whitney U test).

We defined the first survey as ‘Time 1’ and the second survey as ‘Time 2.’ Fig 1 shows the result of the Mann-Whitney U test comparing Time 1 and Time 2. The average scores of GHQ-12 (3.08, 3.73, respectively), IES-R total (6.8, 12.12, respectively), IES-R intrusion (2.31, 4.43, respectively), IES-R avoidance (2.45, 4.16, respectively), and IES-R hyperarousal (2.04, 3.53, respectively) were significantly higher at Time 2 than at Time 1. In other words, Fig 1 suggests that the hospital workers experienced significantly more severe psychological distress 1 year after the pandemic’s beginning.

### Factor analysis

Below are the 26 stress-related questions associated with COVID-19 and the factor analysis results (Table 2). The factor analysis revealed that 20 items loaded on seven factors, with factor loadings ≥0.40 (in **bold**). The seven factors (Factor 1–7), which indicate synthetic variables associated with the stress of the COVID-19 pandemic, were labeled as ‘Isolated,’ ‘Uncertainty,’ ‘Anxiety,’ ‘Exhaustion,’ ‘Workload,’ ‘Feeling of being protected (Protected),’ and ‘Increase of exposure to TV and internet media (TV, internet).’

**Table 2:** Factor analysis of the 26 stress-related questions.

Questions		F1	F2	F3	F4	F5	F6	F7
**Factor 1: Isolated (Cronbach’s α = 0.54)**								
**Q25**	**Unfairness**	**0.613**	-0.054	-0.007	0.008	-0.039	0.058	-0.014
**Q13**	**Hesitation to work**	**0.498**	0.037	0.035	0.023	-0.002	-0.079	0.009
**Q14**	**Feeling of being isolated**	**0.480**	0.060	0.006	0.012	0.112	-0.087	0.003
**Q16**	**Insomnia**	**0.413**	-0.011	-0.022	0.214	-0.064	0.112	-0.028
**Q8**	Feeling of being avoided by others	0.383	0.038	0.037	-0.113	0.031	0.034	-0.004
**Q15**	Elevated mood	0.273	-0.024	-0.013	-0.051	0.015	0.206	-0.036
**Q24**	Greater amount of alcohol drinking	0.257	-0.054	-0.041	-0.017	0.020	0.011	0.141
**Factor 2: Uncertainty (Cronbach’s α = 0.68)**								
**Q7**	**Uncertainty of infectivity and virulence**	-0.094	**0.854**	-0.010	0.007	-0.011	0.021	-0.025
**Q6**	**Uncertainty of means to prevent from infection**	0.006	**0.691**	-0.075	-0.032	0.054	0.032	-0.003
**Q5**	Anxiety of being infected during commuting	0.056	0.360	0.192	-0.041	-0.048	0.044	0.010
**Q26**	Doubt about serious uninformed information	0.212	0.309	-0.021	0.074	-0.067	-0.078	0.030
**Q12**	Anxiety about compensation	0.101	0.249	0.163	0.020	-0.022	-0.077	0.006
**Factor 3: Anxiety (Cronbach’s α = 0.82)**								
**Q1**	**Anxiety about being infected**	-0.003	-0.037	**0.860**	-0.004	-0.009	-0.009	-0.019
**Q2**	**Anxiety about infecting family**	0.013	0.018	**0.832**	-0.023	0.010	0.001	-0.031
**Q21**	Heighten awareness of physical condition management	-0.062	0.081	0.259	0.056	0.012	0.160	0.133
**Factor 4: Exhaustion (Cronbach’s α = 0.84)**								
**Q17**	**Physical exhaustion**	-0.082	-0.006	-0.014	**0.944**	0.020	0.012	-0.013
**Q18**	**Mental exhaustion**	0.138	-0.010	0.008	**0.745**	0.003	-0.019	-0.002
**Factor 5: Workload (Cronbach α = 0.79)**								
**Q3**	**Burden of increase quantity of work**	-0.021	-0.006	-0.038	0.028	**0.862**	0.015	-0.021
**Q4**	**Burden of change of quality of work**	0.034	-0.001	0.040	-0.016	**0.746**	0.001	0.014
**Q20**	Feeling of having no choice but to work due to obligation	0.087	0.067	0.094	0.066	0.131	-0.020	0.057
**Factor 6: Feeling of being protected (Cronbach’s α = 0.50)**								
**Q9**	**Feeling of being protected by national and local governments**	0.072	-0.011	-0.027	-0.039	0.010	**0.683**	-0.010
**Q10**	**Feeling of being protected by the hospital**	0.157	0.018	-0.038	-0.036	-0.018	**0.527**	-0.012
**Q19**	**Motivation to work**	-0.205	0.008	0.068	0.103	-0.001	**0.405**	0.017
**Q11**	Feeling of being supported by family	-0.014	0.048	0.097	0.039	0.042	0.254	0.015
**Factor 7: Increase in exposure to TV and internet media (Cronbach’s α = 0.67)**								
**Q22**	**Increase in TV exposure**	0.047	-0.022	0.003	0.010	-0.018	-0.005	**0.763**
**Q23**	**Increase in internet and SNS exposure**	-0.022	0.013	-0.017	-0.030	0.009	-0.001	**0.664**
	Eigenvalue	4.501	1.218	0.821	0.985	1.069	0.692	0.563
	Variance Explained (%)							37.88
	Between factor correlation							
	F2	0.526						
	F3	0.354	0.591					
	F4	0.585	0.422	0.404				
	F5	0.431	0.389	0.388	.0469			
	F6	-0.222	-0.127	-0.036	-0.110	-0.113		
	F7	0.287	0.308	0.237	0.223	0.189	0.056	

Abbreviation: F, Factor

**Bold**, factor loading **≥ 0.40**

Cronbach’s α was computed without excluded items.

### Multivariable logistic regression analyses

The multivariable logistic regression analyses identified risk factors of the general and/or event-related distress at Time 1 and Time 2. To compare two surveys, risk factors associated with the general distress at Time 1 and Time 2 are presented in Table 3, and those associated with the event-related distress in Table 4.

**Table 3:** Factors associated with severe general distress at Time 1 and Time 2.

	Severe General Distress ^a^			
	Time 1 (March-April 2020) (N = 2644) ^b^		Time 2 (March 2021) (N = 2764) ^c^	
Characteristics and Mental health outcome factors	Adjusted OR ^d^ (95% CI)	P Value	Adjusted OR ^d^ (95% CI)	P Value
Gender, female vs male	1.43 (1.08–1.91)	.013*	1.01 (0.79–1.30)	.933
Age group(years), vs 50-		< .001**		.975
20–29	0.42 (0.30–0.59)	< .001**	0.94 (0.69–1.28)	.676
30–39	0.77 (0.57–1.04)	.092	0.98 (0.73–1.30)	.862
40–49	0.77 (0.58–1.02)	.069	0.99 (0.75–1.30)	.947
Occupation, vs MD		.207		.002*
Nurse	0.91 (0.65–1.28)	.60	1.27 (0.94–1.72)	.119
Other medical professionals ^e^	1.28 (0.89–1.86)	.188	1.88 (1.35–2.60)	< .001**
Office workers and clinical clerks	1.16 (0.81–1.67)	.411	1.50 (1.07–2.11)	.018*
Other support staff ^f^	0.92 (0.58–1.47)	.729	1.15 (0.74–1.77)	.539
Preexisting disease	1.18 (0.81–1.73)	.394	1.13 (0.81–1.57)	.477
Living with partner	0.76 (0.61–0.95)	.014*	0.83 (0.68–1.02)	.077
Living with elderly	0.98 (0.73–1.32)	.901	1.19 (0.89–1.58)	.245
Confident in standard precaution	0.81 (0.66–0.99)	.048*	0.90 (0.75–1.10)	.301
Factor 1 :Isolated	1.67 (1.49–1.87)	< .001**	1.91 (1.72–2.12)	< .001**
Factor 2 :Uncertainty	1.02 (0.90–1.17)	.736	0.94 (0.83–1.07)	.344
Factor 3 :Anxiety	1.12 (0.98–1.29)	.094	1.07 (0.95–1.21)	.286
Factor 4 :Exhaustion	2.76 (2.44–3.12)	< .001**	2.51 (2.22–2.84)	< .001**
Factor 5 :Workload	1.02 (0.90–1.16)	.746	1.05 (0.93–1.17)	.435
Factor 6 :Protected	0.88 (0.76–1.01)	.078	0.84 (0.76–0.93)	.001*
Factor 7 :TV, internet	1.21 (1.08–1.35)	.001*	1.02 (0.91–1.14)	.737
Direct contact with COVID-19 patient	1.02 (0.75–1.40)	.886	1.03 (0.84–1.25)	.800

^a^ Severe General distress evaluated by the GHQ-12 (≥4)

^b^ Data were missing for 53 participants (2.0% of total Time 1 participants)

^c^ Data were missing for 49 participants (1.7% of total Time 2 participants)

^d^ Adjusted for gender, age group, occupation, direct exposure to COVID-19 patients, preexisting disease, living with a partner, living with the elderly, confidence in standard precaution, the factors 1–7 and direct contact with COVID-19 patients.

^e^ Other medical professionals included clinical laboratory technicians, radiological technologists, medical engineers, pharmacists, dieticians, social workers, physical therapists, occupational therapists, and speech therapists.

^f^ Other support staff included nursing assistants, janitors, food service, and laundry staff.

**Table 4:** Factors associated with severe event-related distress at Time 1 and Time 2.

	Severe Event-related Distress ^a^			
	Time1 (March-April 2020) (N = 2644) ^b^		Time2 (March 2021) (N = 2764) ^c^	
Characteristics and Mental health outcome factors	Adjusted OR ^d^ (95% CI)	P Value	Adjusted OR ^d^ (95% CI)	P Value
Gender, female vs male	0.46 (0.27–0.76)	.002*	0.77 (0.55–1.07)	.115
Age group(years), vs 50-		.232		.004*
20–29	0.56 (0.30–1.1)	.075	0.57 (0.39–0.84)	.004*
30–39	0.68 (0.40–1.17)	.162	0.59 (0.41–0.84)	.004*
40–49	0.91 (0.57–1.45)	.693	0.87 (0.63–1.21)	.399
Occupation, vs MD		< .001**		.001*
Nurse	2.36 (1.20–4.70)	.019*	1.12 (0.73–1.70)	.607
Other medical professionals ^e^	1.93 (0.86–4.30)	.11	1.73 (1.11–2.71)	.017*
Office workers and clinical clerks	3.55 (1.75–7.22)	< .001**	1.77 (1.12–2.78)	.014*
Other support staff ^f^	6.42 (2.80–14.7)	< .001**	2.30 (1.34–3.94)	.003*
Preexisting disease	0.96 (0.52–1.77)	.89	1.14 (0.77–1.68)	.506
Living with partner	0.87 (0.59–1.28)	.484	0.89 (0.69–1.14)	.34
Living with elderly	1.02 (0.63–1.65)	.931	1.06 (0.76–1.49)	.721
Confident in standard precaution	1.04 (0.72–1.49)	.854	0.99 (0.78–1.27)	.964
Factor 1 : Isolated	2.13 (1.79–2.54)	< .001**	1.94 (1.72–2.19)	< .001**
Factor 2 : Uncertainty	1.44 (1.12–1.86)	.005*	1.29 (1.11–1.51)	.001*
Factor 3:Anxiety	0.99 (0.74–1.32)	.946	1.09 (0.91–1.30)	.357
Factor 4 : Exhaustion	1.40 (1.05–1.87)	.021*	1.66 (1.36–2.04)	< .001**
Factor 5 : Workload	1.21 (0.97–1.50)	.091	1.05 (0.90–1.23)	.525
Factor 6 : Protected	1.02 (0.80–1.31)	.878	0.93 (0.81–1.06)	.284
Factor 7 : TV, internet	1.26 (1.02–1.55)	.03*	1.07 (0.93–1.23)	.351
Direct contact with COVID-19 patient	0.95 (0.57–1.60)	.852	0.88 (0.68–1.14)	.341
Severe general distress	3.08 (1.96–4.84)	< .001**	2.29 (1.74–3.01)	< .001**

^a^ Severe Event-related Distress evaluated by the IES-R (≥25)

^b^ Data were missing for 53 participants (2.0% of total Time1 participants)

^c^ Data were missing for 49 participants (1.7% of total Time2 participants)

^d^ Adjusted for gender, age group, occupation, direct exposure to COVID-19 patients, preexisting disease, living with a partner, living with the elderly, confidence in standard precaution, the factors1-7, direct contact with COVID-19 patients and severe general distress (evaluated by the GHQ-12).

^e^ Other medical professionals included clinical laboratory technicians, radiological technologists, medical engineers, pharmacists, dieticians, social workers, physical therapists, occupational therapists, and speech therapists.

^f^ Other support staff included nursing assistants, janitors, food service, and laundry staff.

### General distress

As shown in Table 3, female was associated with general distress at Time 1 (OR = 1.43, 95%CI: 1.08–1.91, p = .013) but not at Time 2. Compared to over 50s, 20s showed a significantly lower risk of general distress at Time 1 (OR = 0.42, 95%CI: 0.30–0.59, p < .001) but not at Time 2. Regarding occupations, compared to medical doctors, other medical professionals (OR = 1.88, 95%CI: 1.35–2.60, p < .001) and office workers and clinical clerks (OR = 1.50, 95%CI: 1.07–2.11, p = .018) experienced significantly greater severe general distress at Time 2 but not at Time 1. Those living with a partner (OR = 0.76, 95%CI: 0.61–0.95, p = .014), or confident in standard precaution (OR = 0.81, 95%CI: 0.66–0.99, p = .048) showed a significantly lower risk of general distress than those living without a partner, or not confident in standard precaution, respectively, at Time 1 but not at Time 2.

The ‘Isolated’ and ‘Exhaustion’ were consistent risk factors at both Time1 and Time 2 associated with severe general distress (‘Isolated’ Time 1: OR = 1.67, 95%CI: 1.49–1.87, p < .001, Time 2: OR = 1.91, 95%CI: 1.72–2.12, p < .001; ‘Exhaustion’ Time 1: OR = 2.76, 95%CI: 2.44–3.11, p < .001, Time 2: OR = 2.51, 95%CI: 2.22–2.84, p < .001). ‘TV, Internet’ were associated with general distress at Time 1 (OR = 1.21, 95%CI: 1.08–1.35, p = .001) but not associated at Time 2. ‘Protected’ showed a significantly lower risk of severe general distress only at Time 2 (OR = 0.84, 95%CI: 0.76–0.93, p = .001).

### Event-related distress

Table 4 shows the results of the multivariable logistic regression analyses that identified risk factors of severe event-related distress at Time 1 and Time 2. In contrast to the general distress, females showed a significantly lower risk of event-related distress than males at Time 1 (OR = 0.46, 95%CI: 0.27–0.76, p = .002) but not at Time 2. Among age groups, although the association at Time 1 was non-significant, 20s and 30s showed a significantly lower risk of event-related distress at Time 2 compared to over 50s (20s: OR = 0.57, 95%CI: 0.39–0.84, p = .004; 30s: OR = 0.59, 95%CI: 0.41–0.84, p = .004). Regarding occupations, compared to medical doctors, nurses (OR = 2.33, 95%CI: 1.15–4.70, p = .019), office workers and clinical clerks (OR = 3.55, 95%CI: 1.75–7.22, p < .001), and other support staff (OR = 6.42, 95%CI: 2.80–14.7, p < .001) had significant severe event-related distress at Time 1, whereas other medical professionals (OR = 1.73, 95%CI: 1.11–2.71, p = .017), office workers and clinical clerks (OR = 1.77, 95%CI: 1.12–2.78, p = .014) and other support staff (OR = 2.30, 95%CI: 1.34–3.94, p = .003) showed significant distress at Time 2.

The ‘Isolated,’ ‘Uncertainty,’ and ‘Exhaustion’ were consistent risk factors at Time1 and Time 2 associated with severe event-related distress (‘Isolated’ Time 1: OR = 1.67, 95%CI: 1.49–1.87, p < .001, Time 2: OR = 1.91, 95%CI: 1.72–2.12, p < .001; ‘Exhaustion’ Time 1: OR = 2.76, 95%CI: 2.44–3.11, p < .001, Time 2: OR = 2.51, 95%CI: 2.22–2.84, p < .001). ‘TV, Internet’ were associated with general distress at Time 1 (OR = 1.21, 95%CI: 1.08–1.35, p = .001) but not at Time 2. ‘Uncertainty’ and severe general distress were risk factors for severe event-related distress at Time 1 and Time 2.

### Suicide-related ideation

Factors associated with suicide-related ideation identified by multivariable logistic regression analysis are shown in Table 5. Gender and occupations were not significantly associated with suicide-related ideation. Among age groups, compared to participants in their 50s, those in their 20s (OR = 2.34, 95%CI: 1.39–3.96, p = .001), 30s (OR = 3.01, 95%CI: 1.83–4.94, p < .001), and 40s (OR = 2.04, 95%CI: 1.25–3.32, p = .004) showed higher risks for suicide-related ideation.

**Table 5:** Factors associated with suicide-related ideation identified by multivariable logistic regression analysis.

	Suicide-related Ideation ^a^ (N = 2764) ^b^	
Characteristics and Mental health outcome factors	Adjusted OR ^c^ (95% CI)	P Value
Gender, female vs male	1.23 (0.81–1.88)	.335
Age group(years), vs 50-		< .001**
20–29	2.34 (1.39–3.96)	.001*
30–39	3.01 (1.83–4.94)	< .001**
40–49	2.04 (1.25–3.32)	.004*
Occupation, vs MD		.17
Nurse	0.70 (0.42–1.17)	.171
Other medical professionals ^d^	1.06 (0.62–1.82)	.839
Office workers and clinical clerks	0.77 (0.44–1.36)	.369
Other support staff ^e^	1.32 (0.64–2.69)	.452
Preexisting disease	0.91 (0.53–1.53)	.711
Living with partner	1.33 (0.96–1.83)	.088
Living with elderly	1.03 (0.66–1.61)	.89
Confident in standard precaution	1.19 (0.88–1.61)	.271
Factor 1: Isolated	1.47 (1.28–1.70)	< .001**
Factor 2: Uncertainty	1.07 (0.87–1.31)	.532
Factor 3: Anxiety	0.67 (0.54–0.84)	< .001**
Factor 4: Exhaustion	1.11 (0.85–1.44)	.457
Factor 5: Workload	0.96 (0.79–1.17)	.672
Factor 6: Protected	0.85 (0.71–1.03)	.09
Factor 7: TV, internet	1.14 (0.95–1.37)	.168
Direct contact with COVID-19 patient	0.65 (0.47–0.90)	.011*
Severe general distress ^f^	6.20 (3.90–9.86)	< .001**
Severe event-related distress ^g^	2.45 (1.75–3.43)	< .001**

^a^ Suicide-related ideation evaluated by the score of Item 9 of PHQ-9 (≥ = 1).

^b^ Data were missing for 49 participants (1.7% of total participants).

^c^ Adjusted for gender, age group, occupation, preexisting disease, living with a partner, living with the elderly, confident in standard precaution, the factors1-7, direct contact with COVID-19 patients, General distress and Event-related distress.

^d^ Other medical professionals included clinical laboratory technicians, radiological technologists, medical engineers, pharmacists, dieticians, social workers, physical therapists, occupational therapists, and speech therapists.

^e^ Other support staff included nursing assistants, janitors, food service, and laundry staff.

^f^ Severe general distress evaluated by the GHQ-12 (≥4)

^g^ Severe event-related distress evaluated by the IES-R (≥25)

Similar to general and event-related distress, the ‘Isolated’ was significantly associated with suicide-related ideation. ‘Anxiety’ (OR = 0.67, 95%CI: 0.54–0.84, p < .001) and direct contact with a COVID-19 patient (OR = 0.65, 95%CI: 0.47–0.90, p = .011) showed a significantly lower risk of suicide-related ideation. Both severe general distress (OR = 6.20, 95%CI: 3.90–9.86, p < .001) and severe event-related distress (OR = 2.45, 95%CI: 1.75–3.43, p < .001) were risk factors for suicide-related ideation.

## Discussion

Our survey studies showed that the psychological health of hospital workers had deteriorated over 1-year after the beginning of the COVID-19 pandemic. The number of hospital workers who reported severe general and/or event-related distress increased from March-April 2020 to March 2021, and the mean scores of GHQ-12 and IES-R had escalated. Our results showed that more than 8% of the hospital workers experienced suicide-related ideation 1-year after the pandemic’s beginning. The ‘Isolated’ and the ‘Exhaustion’ were consistent risk factors at Time 1 and Time 2 associated with both severe general and event-related distress. The ‘Uncertainty’ was a consistent risk factor at Time 1 and Time 2 for event-related distress. The severe general and event-related distress, and the ‘Isolated’ were risk factors for suicide-related ideation. Our results indicated that ‘Anxiety’ and direct contact with COVID-19 patients were protective factors for suicide-related ideation.

This study demonstrated that the psychological distress among the hospital workers had significantly deteriorated over 1-year after the beginning of the COVID-19 pandemic. Many analyses have shown increased global prevalence and burden of depressive and anxiety disorders worldwide due to the COVID-19 pandemic [4, 38]. In particular, a considerable percentage of HCWs reported serious psychiatric symptoms during the COVID-19 pandemic [9, 39], including HCWs in our previous study [19]. Furthermore, several longitudinal studies have reported that psychological distress, anxiety, and depression, and fear and worry about COVID-19 increased statistically significantly more among HCWs than non-HCWs [17, 21]. Our findings are consistent with other studies and provide evidence for the negative psychological effects of the COVID-19 pandemic and their change from the beginning of the pandemic to one year after among hospital workers.

Our studies showed that the ‘Isolated’ was a risk factor for both general and event-related distress at Time 1 and Time 2, and also for suicide-related ideation at Time 2. In our previous analysis [19], although the risk factors for general psychological distress and event-related distress were evaluated separately for high- and low-risk workers for infection, the ‘Feeling of being isolated and discriminated’ was also a common risk factor for general and event-related distress of workers both high- and low-risk for infection. Our results at the beginning of the COVID-19 pandemic were consistent with other studies on the mental health of HCWs during the SARS pandemic, one of the most major pandemics in recent decades. These studies reported that the hospital workers felt “being treated differently because of working in hospital” [40] or “people avoid my family members because of my job,” [41]. One year after the beginning of the COVID-19 pandemic, many people had already been accustomed to COVID-19; however, our results showed that the psychological distress of the hospital workers deteriorated further, and “Isolation” remained a risk factor for psychological distress.

We showed that ‘uncertainty’ was a consistent risk factor for event-related distress at Time 1 and Time 2. This result is in line with many previous analyses showing that uncertainty intolerance directly affected mental well-being during the COVID-19 pandemic [42–44]. Moreover, other analyses have found that intolerance of uncertainty correlated with increased suicidal ideation [45, 46].

In our study, the number of hospital workers who reported severe general distress measured by GHQ-12 and/or event-related distress measured by IES-R increased, and the mean scores of both GHQ-12 and IES-R among all participants escalated from Time 1 to Time 2. In addition, severe general and event-related distress were risk factors for suicide-related ideation at Time 2. Although we did not assess suicide-related ideation in the first survey, whether suicide-related ideation increased is uncertain, although our results showed that more than 8% of the hospital workers experienced suicide-related ideation in March 2021. Other studies have reported the prevalence of suicide-related ideation based on Item 9 of PHQ-9 ≥1 as in our study, which was 6.7% from May to June 2020 among Japanese university students [33] and 5.4% among HCWs in the U.S. in April 2020 [20]. In our analyses, the higher levels of psychological distress were associated with suicide-related ideation.

Our results showed that ‘anxiety’ and direct contact with COVID-19 patients were protective factors for suicide-related ideation. Previous studies have reported that anxiety correlates independently with depression and suicide-related ideation [47–49]. However, a previous study on HCWs and medical students in Japan showed that the severity of depression symptoms was not correlated with anxiety symptoms [50]. Although it may be essential to differentiate anxiety disorder from anxiety symptoms or ‘anxiety’ from COVID-19 infection here in our analysis, our analysis showed that recognizing or expressing ‘anxiety’ from infection could be a protective factor for suicide-related ideation. Since HCWs were described in heroic terms during the COVID-19 pandemic, they may have experienced a fear of stigma when expressing anxiety about infection openly and seeking mental health care [16]. Direct contact with the COVID-19 patients may have become a protective factor for the suicide-related ideation because it may have diminished some of the uncertainty of COVID-19 by actually experiencing it. Thus, direct contact with COVID-19 patients may indirectly reduce event-related distress and could be a protective factor for suicide-related ideation.

It must be noted that this study had some limitations, though. First, the hospital workers sampled during the first and second surveys were not the same because of the transfer, new employment, or resignation. Due to the anonymous data collection, we could not pair respondents at 1-year intervals. Second, because we did not assess suicide-related ideation in the first survey, it is unclear whether it increased.

Unlike at the beginning of the pandemic, when we didn’t know what it was and how to cope with it, many people now have a basic knowledge of COVID-19 and are accustomed to it. In contrast, as we write this manuscript in July 2022, the seventh wave with the largest number of infected people on record has come to Japan. Uncertainty about the future continues due to differences in infectivity and changes in social adaptation. The evidence from the SARS pandemic indicates that HCWs’ psychological distress can persist for up to 3 years after an outbreak has ended [51, 52]. Unlike the previous pandemic, the COVID-19 pandemic is prolonged, affects the entire world, and its psychological effects are unpredictable. The COVID-19 situation has continued to change after our research, and infectious situations and social adaptations vary across countries and periods; hence, we are planning to continually investigate the psychological effect of HCWs during the COVID-19 pandemic.

## Conclusion

Our survey studies showed that mental health outcomes among the hospital workers had deteriorated over one year from the pandemic’s beginning, and their severe psychological distress was the risk factor for suicide-related ideation. Further studies are needed to compare the psychological effect of COVID-19 on hospital workers and to explore appropriate measures to support hospital workers’ mental health.
